# First implementation and results of online adaptive radiotherapy for cervical cancer based on CT-Linac combination

**DOI:** 10.3389/fonc.2024.1399468

**Published:** 2024-11-14

**Authors:** Dazhen Jiang, Chunxu Yang, Shaoxing Sun, Dajiang Wang, Zhizhi Xiao, Jie Hu, Zijie Mei, Conghua Xie, Hui Liu, Hui Qiu, Xiaoyong Wang

**Affiliations:** Department of Radiation and Medical Oncology, Hubei Key Laboratory of Tumor Biological Behaviors, Hubei Cancer Clinical Study Center, Zhongnan Hospital of Wuhan University, Wuhan, China

**Keywords:** online adaptive radiation therapy, cervical cancer, FBCT-Linac, image-guide radiation therapy, dosimetry, survival follow-up

## Abstract

**Purpose:**

To assess the dosimetric effectiveness of image-guided radiation treatment (IGRT) and online adaptive radiation therapy (oART) for cervical cancer. As well as survival follow-up was conducted to validated the safety and efficacy of oART.

**Methods:**

A total of 15 cervical cancer patients were enrolled. oART was performed on a CT-integrated linear accelerator. The initial plan was revised to include the distribution of IGRT dose using daily fan-beam CT (FBCT) images, after which the distinctions between ART and IGRT in terms of target coverage and organs at risk (OARs) sparing were analyzed. Survival follow-up was conducted to validated the safety and efficacy of oART in this group.

**Results:**

PTV Dmax value decreased by 1.23 Gy in the ART plan when compared to that in the IGRT plan; PTV D95 increased by 1.34 Gy; PTV V50 coverage increased by 4.86%; CTV coverage increased by 3.02%; PTV D2cc of the colon, rectum, and small intestine decreased by 1.24 Gy, 1.29 Gy, and 1.12 Gy, respectively. The V10 and V30 of the pelvis increased by 1.27% and 0.56%, respectively, while the V30 of the left and right femoral heads dropped by 2.82% and 3.41%, respectively. Except for the pelvic changes, all other differences were statistically significant (p < 0.01). The average time for the ART procedure was 21.22 min (range: 18.72–24.90 min). The median follow-up time is 28.0 months. Median event-free survival and overall survival were not reached. EFS rate and OS rate at 3 years were 79.4% and 92.9%.

**Conclusion:**

Online ART for cervical cancer can minimize the dose of OARs and enhance the target volume coverage significantly when compared to IGRT with satisfied survival time.

## Introduction

Globally, the fourth most prevalent malignancy in women is cervical cancer (1). For the past 15 years, intensity-modulated radiotherapy (IMRT) and image-guided radiation (IGRT) have been used as modifications to the external beam radiation treatment for gynecological cancer. IGRT has made personalized treatment (2, 3), dose escalation, better clinical outcomes, and less toxicity to normal tissues (4, 5) possible. The capacity to adhere to target quantities, which decreases the dose administered to organs at risk (OARs), also motivates the clinical use of these modifications (6). Clinical improvements are evident as a decrease in acute and chronic gastrointestinal and hematological toxicities have been observed with the switch from pelvic three-dimensional conformal radiation (3DCRT) to IMRT (7, 8). The risk of geographical miss arising from the complex and varying inter- and intrafractional mobility observed with the cervix-uterus complex was an initial cause of concern for the application of IMRT for gynecological cancer treatment (9). This depends on tumor regression during radiation therapy and bladder and rectal filling (9, 10). A mean interfractional cervical motion of 2.3–16 mm in the anterioposterior direction, 2.7–8 mm in the superior–inferior direction, and 0.3–10 mm in the lateral direction was reported in a systematic review (11). According to Tyagi et al. (12), a margin of >35 mm would be necessary to cover the clinical target volume (CTV) for every fraction. However, one-third of the cases failed to cover the CTV as a 15-mm margin was used. Cervical cancer treatment needs to be more personalized so as to address this interpatient variability. However, due to the special anatomical position of the cervix, adjacent to the bladder and rectum with obvious changes in physiological status (varying due to the degree of filling), coupled with random factors such as tumor retraction, the target area may be deformed and moved between fractional treatments, which may lead to dose distribution deviation.

Online adaptive radiotherapy (online ART) is another development in adaptive radiation therapy (ART) methods. OART can modify plans based on real-time images and can be executed immediately to accommodate random transfer of target volume (TV) and organs, as well as the deformation of OARs in the fractional treatments, to ensure the coverage of the target dose and to control the dose of OARs (13–15). With daily replanning and oART, the issue of interfractional mobility may be reduced. Ten years’ worth of technological and computational developments in radiotherapy have thankfully made oART a clinical potential (13, 15). This therapeutic approach relies on daily anatomical fluctuations in target volumes and OARs and changes in tumor volume. Therefore, planning treatment volume (PTV) margins may decrease (16). Most commercially available oART systems are guided by magnetic resonance imaging (MRI); however, recent developments have resulted in the development of a cone beam computed tomography (CBCT)-guided system. **Table 1** presents the possible advantages of oART in cervical cancer external beam radiation treatment with CBCT and MRI guidance.

**Table 1:** Patients’ characteristics.

	N	%
Total	15	100%
Age (Median, range)	61 (42-65)	
Diagnosis		
Cervical cancer	14	93.3%
Ovarian cancer	1	6.7%
Stage at time of diagnosis		
IB1	1	6.7%
IB3	1	6.7%
IIA1	2	13.3%
IIA2	1	6.7%
IIB	1	6.7%
IIIB	1	6.7%
IIIC1	3	20.0%
IIIC2	1	6.7%
IVa	3	20.0%
IVb	1	6.7%
Disease status at time of ART		
Newly diagnosed	13	86.7%
Local recurrent disease	1	6.7%
Metastasis disease	1	6.7%
Prior treatment lines		
0	5	33.3%
1	9	60.0%
≥2	1	6.7%
RT purpose		
Adjuvant RT	3	20.0%
Palliative RT	2	13.3%
Radical RT	10	66.7%
Radiated body parts		
Pelvic only	14	93.3%
Pelvic and lower abdominal	1	6.7%
Duration of external beam radiation (days) (Median, range)	44 (36-59)	
Brachytherapy		
Yes	12	80.0%
No	3	20.0%
Concurrent chemotherapy		
Yes	10	66.7%
No	5	33.3%
Prescription dose (Point A) (EQD2) (Median, range)		
External beam radiation	44.25 (44.25-50)	
Brachytherapy	40 (16-40)	

The currently used oART is based on transforming CBCT and MRI images into pseudo-CT images for radiotherapy planning design, which increases the time of oART and introduces certain errors. The FBCT-linac accelerator, a fan-beam computed tomography (CT), which may be used directly in radiotherapy planning design, is the basis for the present investigation. This accelerator considerably shortens the time of oART and improves the dose accuracy.

This study compared oART and a clinically implemented plan selection technique to quantify the added value of oART performed for cervical cancer by using the FBCT-linac accelerator. The benefit was measured in terms of target volume coverage and dose to OARs. The aim of this study was to evaluate the long-term clinical results of oART in a single center and contribute to the rare evidence in that field.

## Methods

### Patient selection and simulation

We enrolled 15 cervical cancer patients received pelvic IMRT treatment at our facility between January 2022 and November 2022 in this study. According to FIGO stage classification (17), the patients information were presented in **Table 1**. A 3-mm slice thickness helical CT scanner (Sensation Cardiac 64x, Siemens, Munich, Bavaria, Germany) was used to perform CT simulation. Before the simulation was started, the patients were placed in a supine position, and a vacuum-formed cradle, a comfortably full bladder, and no bowel preparation were ensured.

15 patients radiated with ART technique were enrolled. 14 of them were diagnosed as cervical cancer. 10 received radical concurrent chemoradiation and 3 received adjuvant radiotherapy. The median prescription dose at point A was 44.25Gy for external beam radiation. 12 of them received brachytherapy with median dose of 40Gy. Patients characteristics were listed in **Table 1**.

### Normal tissue definition

In this study, the rectum, small bowel, bladder, femoral heads, and bone marrow were all considered normal tissue. The small bowel, which encompasses the entire peritoneal cavity from L4 to L5, was contoured for each patient. This contour, along with the others, was defined by a single radiation oncologist so as to ensure consistency in target definition and treatment planning. GTVp refers to the primary lesion of cervical tumors. GTVn refers to pelvic metastatic lymph nodes. From the L4-L5 vertebra to the inferior border of the obturator foramen, CTV generally includes vaginal stumps, common iliac, internal iliac, external iliac, obturator and presacral lymphatic drainage areas, cervix, uterine body, parauterine and partial vagina. Delineations method of target volumn complied with ICRU report 50 and 62 and consensus guidelines developed by RTOG (18). The PTV was produced by applying a uniform 5-mm margin to the CTV (19).

**Figure 1:**
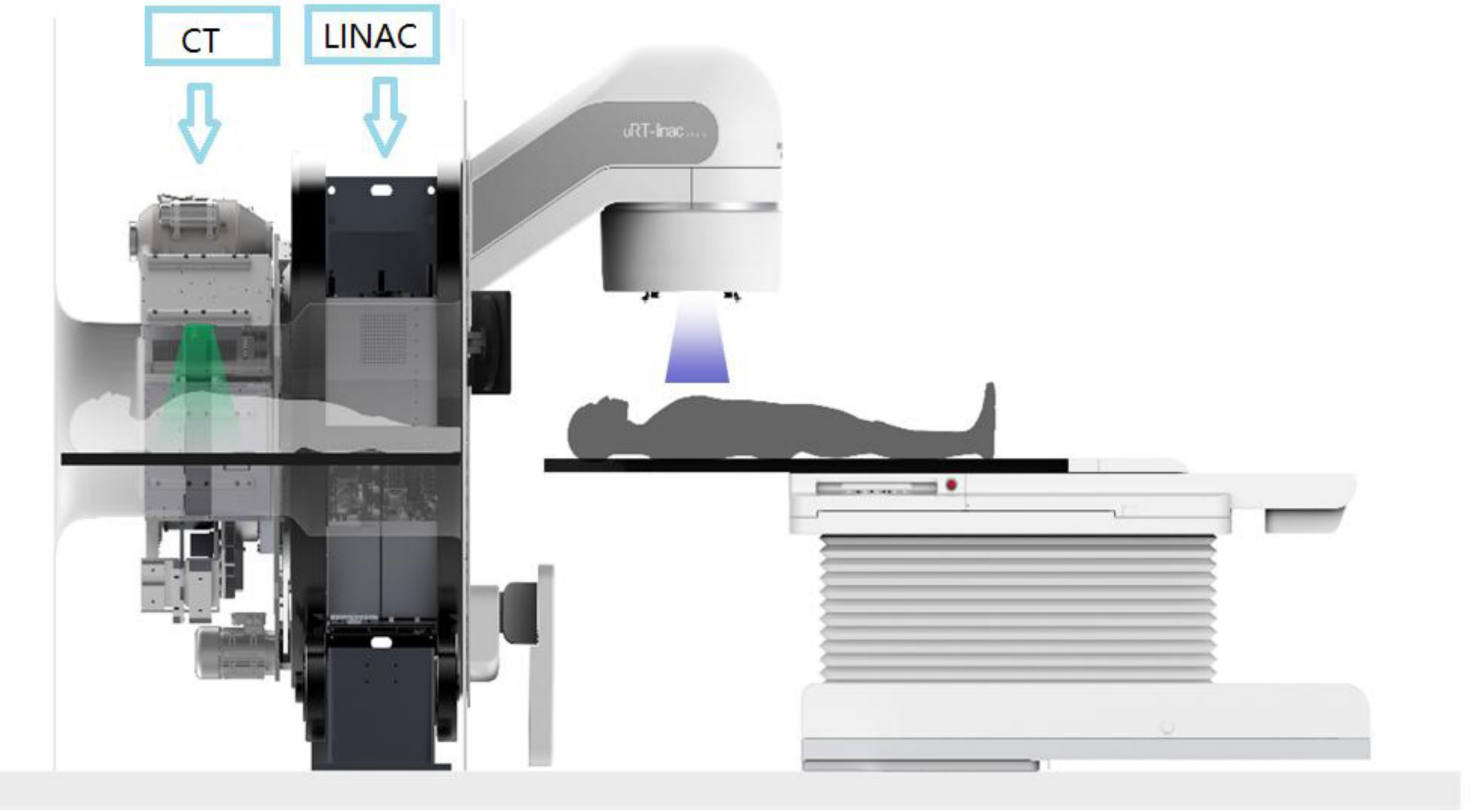
The linear accelerator of United Imaging Healthcare’s CT linac URT-Linac 506C.

To ensure consistency, each treatment planning technique was created by the same radiation physicist. **Figure 1** illustrates how United Imaging TPS (United Imaging HealthCare Co., LTD, Shanghai, China) was used to design the inverse planned dynamic IMRT plans. Using the analytic CC algorithm with a grid size of 2.0 mm, the dosage to be administered to the irradiated zone was calculated. With 6 MV photon beams, sliding-window fields, and a multileaf collimator leaf size of 5 mm, we applied the machine parameter optimization approach.

### IMRT therapy planning

By using the initial radiotherapy CT scans of all patients, a radiation treatment plan was designed at the United Imaging FBCT-Linac workstation for each patient. This plan was prescribed as a 25-day IMRT session. A 9-field coplanar static intensity-modulated radiation treatment plan was implemented with a 50-Gy prescribed dose for PTV-CTV.

### Online ART plan

After the patient was positioning, the FBCT images were acquired based on the oART process. The simulation CT was registered to the FBCT by using a deformable alignment. Normal organs were delineated by TPS based on the outputs of the coimage automatic contour system. An experienced clinician modified the CTV after deformable registration. The PTV was generated by expanding the 5-mm margin from the GTV and CTV. Based on FBCT, oART plans were automatically generated using TPS algorithms and were evaluated by physicists and clinicians. After the plans were evaluated, they were transmitted to the treatment terminal for FBCT guidance and treatment.

### Evaluation indicators

The plans of patients receiving IGRT and ART must be assessed for the actual target coverage rate and OAR sparing. The IGRT parameter values can be obtained as follows. The treatment plan for the simulation FBCT was first strictly copied to the FBCT on each treatment day to calculate the dose as the IGRT dose received by the patient in each treatment fraction. The dose parameters of each IGRT plan were statistically analyzed, and the final average IGRT values were then obtained. Similarly, the final average ART values.

The dose and coverage of PTV and OARs were compared. Dx% was the maximum dose of volume accepted by x% of PTV or OARs, and Vx% was the volume of the prescribed dose line for x% of PTV or OARs. The plan had to be approved with at least 95% of the PTV receiving 100% of the prescribed dose, and the PTV’s maximum dosage had to be <110% of the prescribed dose. Some dosimetric parameters were collected for the OARs, namely small bowel, rectum, femoral heads, bone marrow, and colon sigmoid, including the V30 (the percent of volume that received 30 Gy) for the femoral heads and bone marrow. These values also included the maximum dose of 2 cc (D2cc) of the structure representing the significant maximum dosage for the colon sigmoid, rectum, and small bowel.


$$In\;this\;study,\;\text{difference}=\left(\frac{Values_{ART}-Values_{IGRT}}{Values_{ART}}\right)*100\%$$

### Statistical analysis

The Statistical Package for Social Sciences (version 22.0, IBM Corporation, Armonk, NY, USA) was used for data analysis. The Shapiro−Wilk method was used to test the normality of the data. A paired sample t test was performed for data lines with a normal distribution, while the Wilcoxon signed rank test was performed with a significance level of 0.05.

## Results

**Table 2** presents all values. Compared with those of the IGRT plan, the PTV Dmax of the ART plan decreased by 1.23 Gy; its PTV D95 increased by 1.34 Gy; its PTV coverage V50 increased by 4.86%; its CTV coverage increased by 3.02%; and its D2cc of the small intestine, rectum, and colon decreased by 1.24 Gy, 1.29 Gy, and 1.12 Gy, respectively. The V30 of the left and right femoral heads decreased by 2.82% and 3.41%, respectively. The V10 and V30 of the pelvis increased by 1.27% and 0.56%, respectively. Except for the pelvis, statistically significant differences were observed for all other organs (P < 0.01).

Table 2Depicts the parametric statistics of PTV, CTV, and each OAR.

**Table:** 

		PTV Dmax*	PTV D95*	PTV V50*	CTV v50*
Dmean	ART	5334.55	5030.11	97.29	99.63
	IGRT	5458.30	4895.69	92.43	96.60
Standard deviation	ART	42.581	18.47	0.58	0.20
	IGRT	45.02	32.66	1.22	0.65

		D2cc			V30			V10
		Colon*	Small bowel*	Rectum*	Pelvic bone	Left femoral head*	Right femoral head*	Pelvic bone
Dmean	ART	5185.96	5207.81	5170.10	48.69	3.08	3.69	85.66
	IGRT	5298.31	5331.83	5299.57	49.97	5.90	7.10	86.22
Standard deviation	ART	7.99	8.92	22.53	1.43	0.74	0.79	1.52
	IGRT	54.02	69.65	63.57	0.30	1.12	0.91	1.55

where * represents a statistical difference between the two groups.

D2cc refers to the statistical parameter for the colon, small intestine, and rectum, while V30 is the parameter specifically used for the femoral head and pelvic bone, V10 is the parameter specifically used for the pelvic bone.

**Table 3** presents the average time taken for imaging, contouring, planning, delivery and the time it takes for patients to enter and leave the treatment room.

**Table 3:** Overview of steps and duration.

Time (seconds) needed for	Average	Min	Max
Position validation CT (FBCT)	107	58	164
Generate PTV and OARs	125	102	193
Evaluation and adjustment target	257	189	335
Plan calculation	294	181	396
Treatment delivery	368	322	445
Patient entering and leaving treatment room	1273	1122	1494

**Figure 2:**
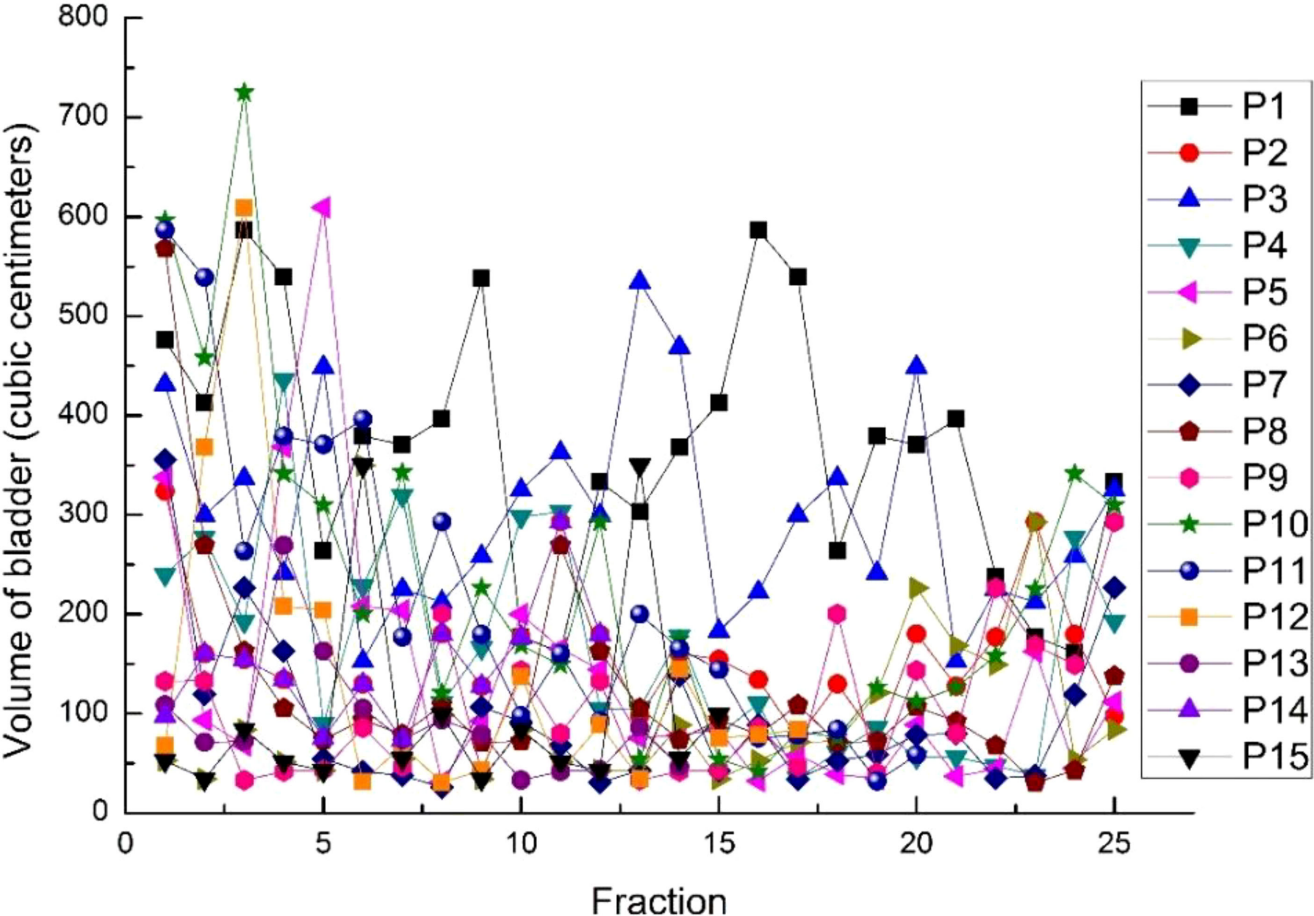
The volumes of the bladder in 15 cases for every fraction.

From **Figure 2**, we can see that the changes among fractional treatments were irregular. However, it can be seen that as the number of treatments increases, the patient’s urine retention decreases, which may be due to a certain degree of cystitis.

**Figure 3:**
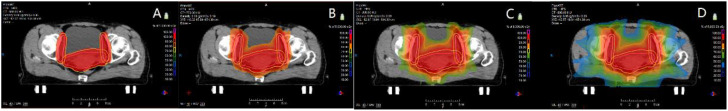
The ART plan dose distribution for one of the patients with **(A)** 100% (50 Gy), **(B)** 80% (40 Gy), **(C)** 60% (30 Gy) and **(D)** 40% (20 Gy) on coronal views.

**Figure 4:**
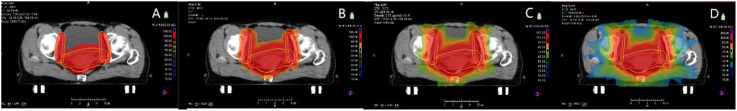
The IGRT plan dose distribution for one of the patients with **(A)** 100% (50 Gy), **(B)** 80% (40 Gy), **(C)** 60% (30 Gy) and **(D)** 40% (20 Gy) on coronal views.

From **Figures 3**, **4**, we can see that compared to the IGRT plan, the ART plan can significantly improve the coverage of the target area while better protecting organs at risk.

**Figure 5:**
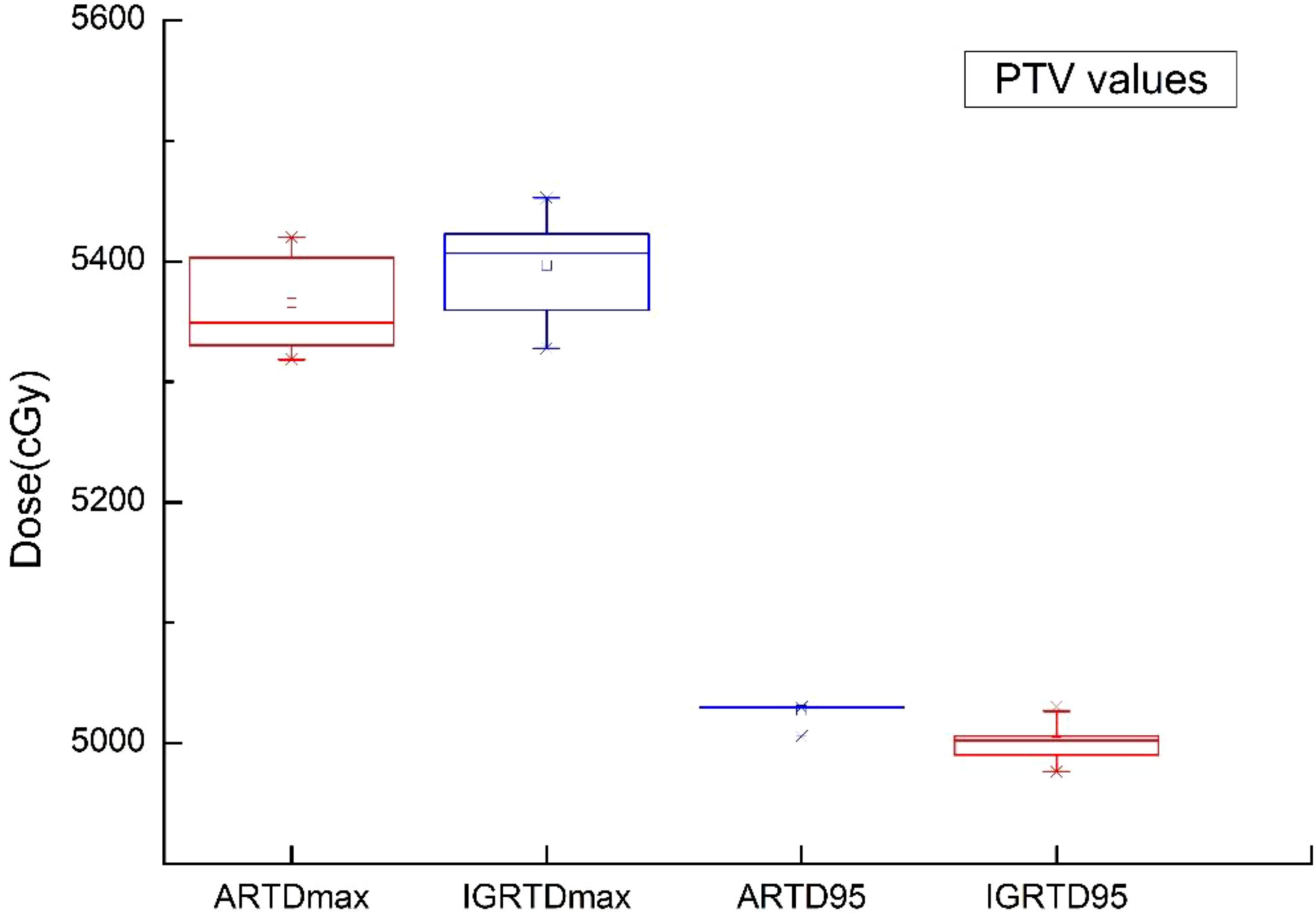
Boxplot showed the values of PTV different DVH parameters for both IGRT selection and online ART.

**Figure 6:**
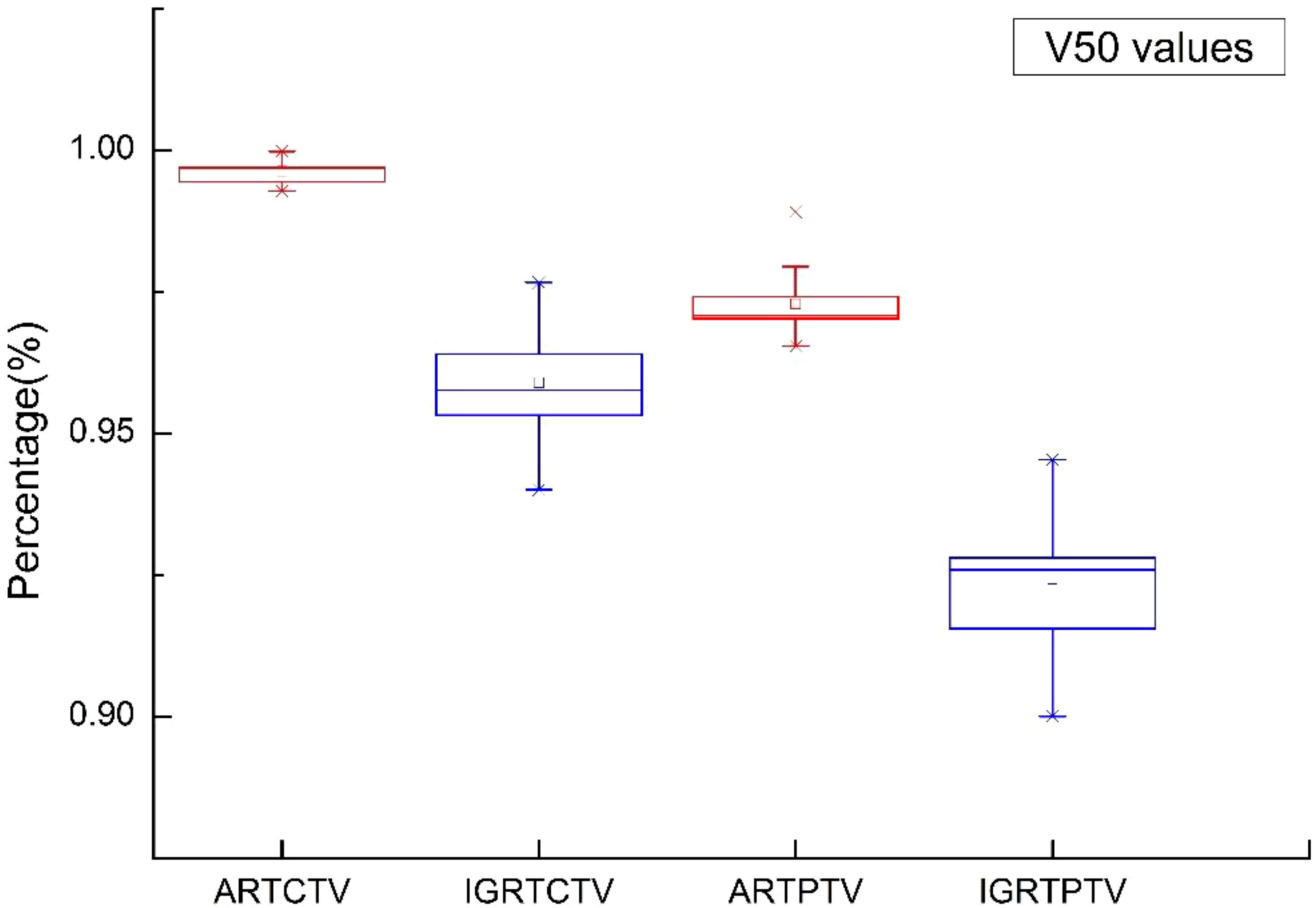
Boxplot showed the coverage rate of PTV different DVH parameters for both IGRT selection and online ART.

**Figure 7:**
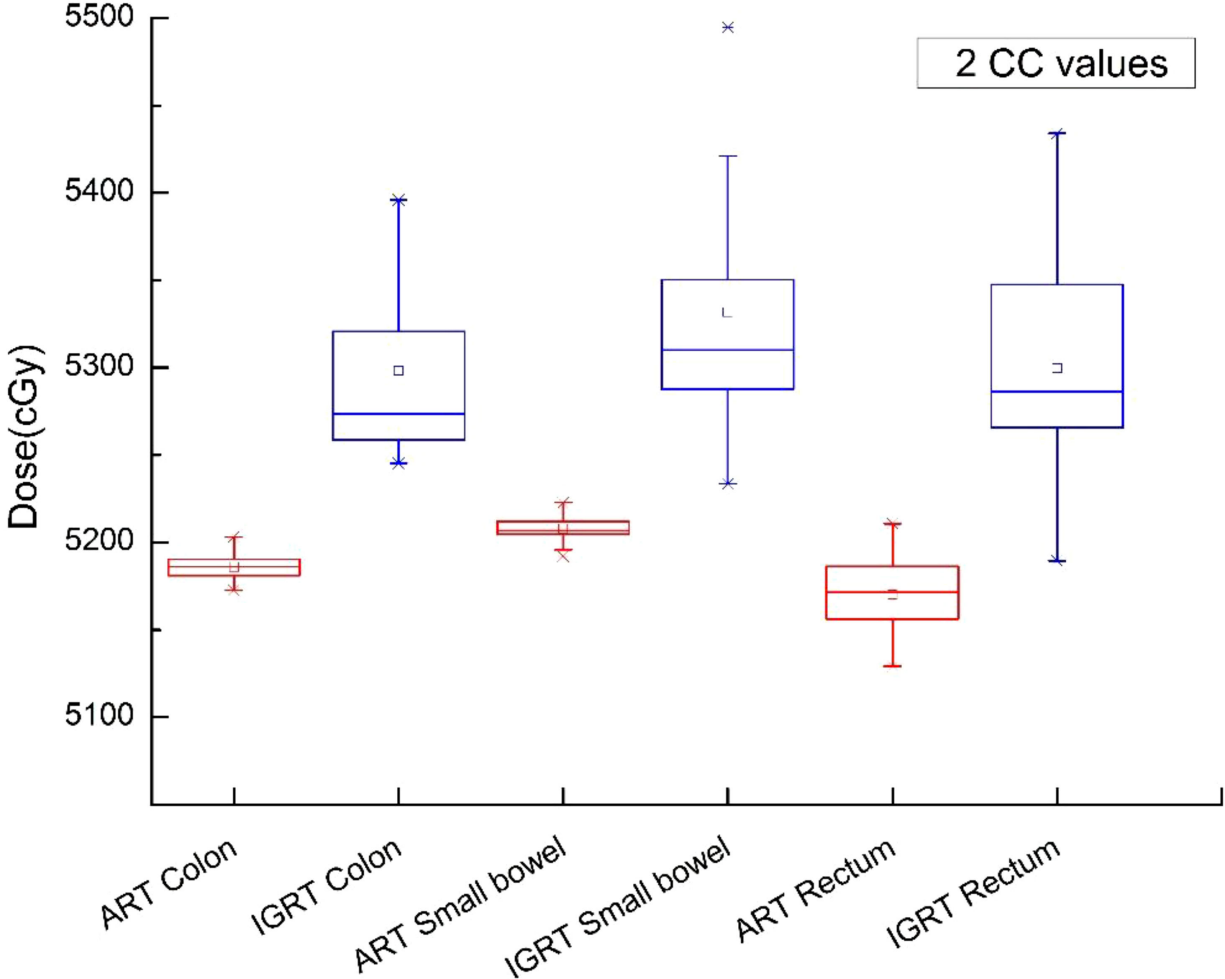
Boxplot showed the 2cc values of OAR different DVH parameters for both IGRT selection and online ART.

**Figure 8:**
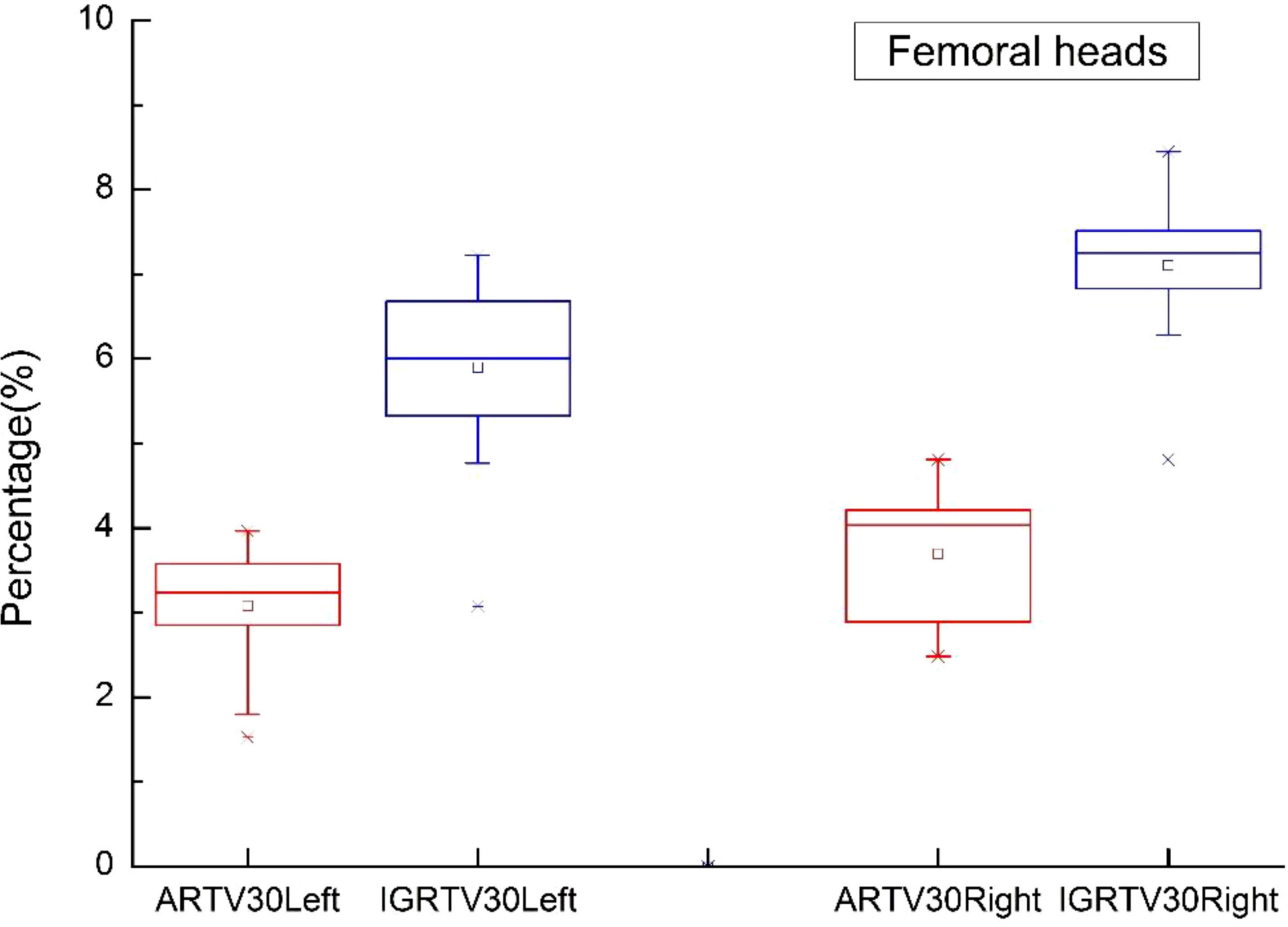
Boxplot showed the values of femoral heads different DVH parameters for both IGRT selection and online ART.

**Figure 9:**
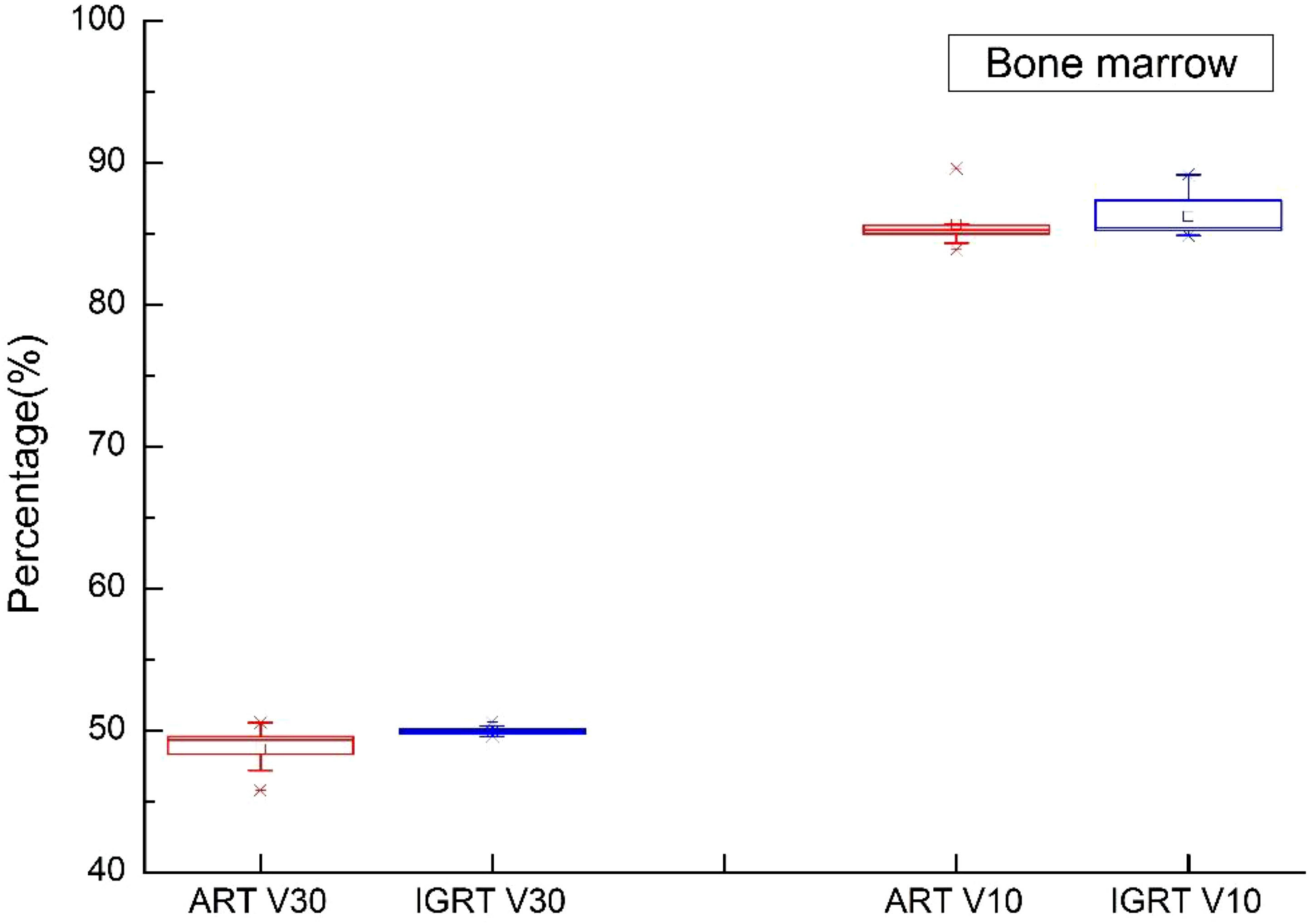
Boxplot showed the values of bone marrow different DVH parameters for both IGRT selection and online ART.

In this study, **Figures 5**–**9** present PTV and OAR DVH parameters for both IGRT selection and oART. Compared with the IGRT plan, target coverage rate parameters for the ART plan were improved. Significant differences in V50 and D95 (P < 0.05) were observed for PTV parameters between the two groups. The V50 values of GTV significantly differed between the two groups (P < 0.05). For the colon, rectum, and small intestine, the D2cc values of IGRT were higher than those of ART.

In this study, the average time from positioning to the end of beam exit was 21.22 minutes (SD 2.057 minutes), with an average positioning time of 107 seconds (SD 26.60 seconds), target modification time of 257 seconds (SD 59.21 seconds), rescheduling time of 294 seconds (SD 57.32), and beam exit time of 368 seconds (SD 64.01).

**Figure 10:**
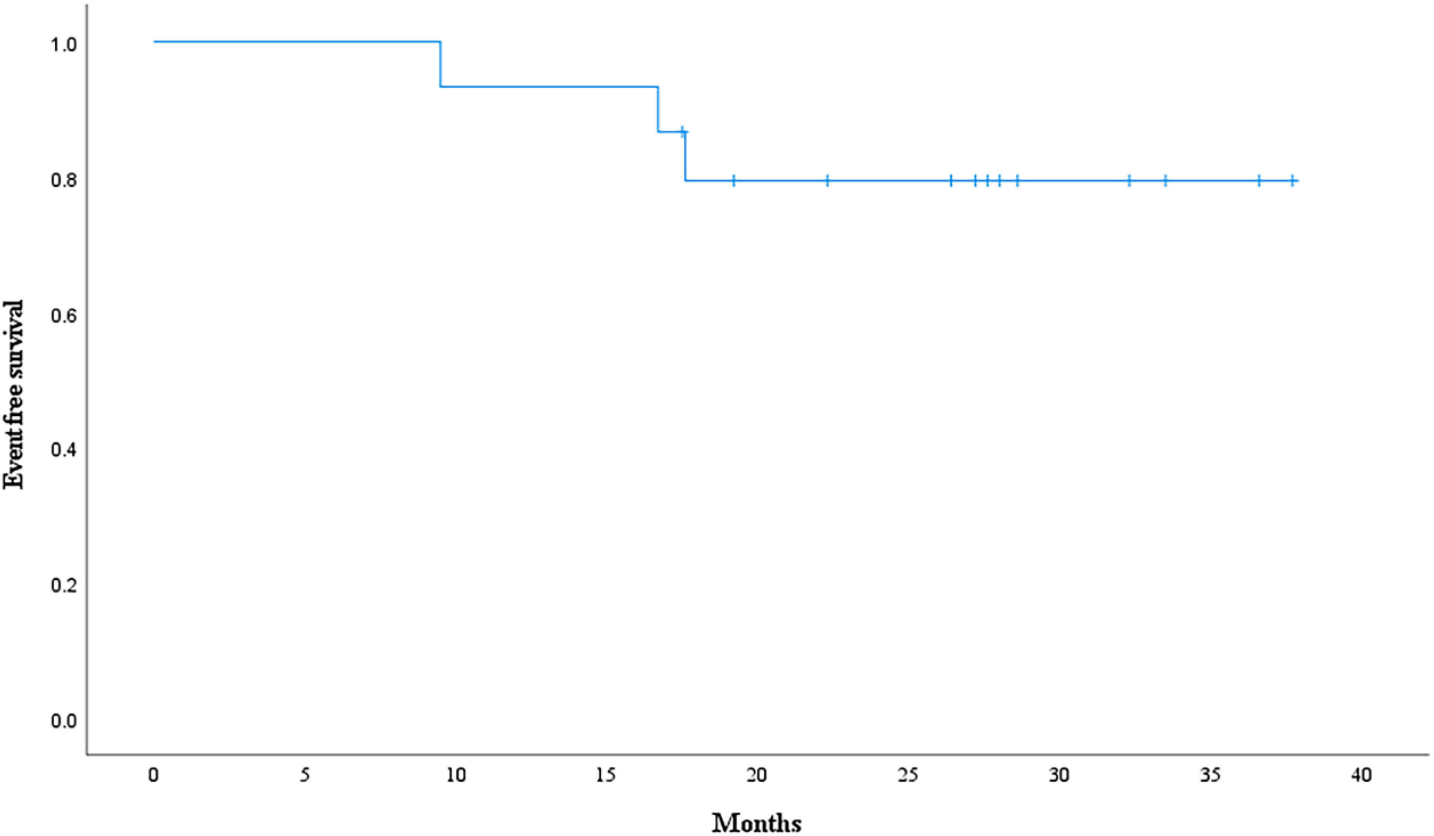
Event-free survival curve calculated with Kaplan-Meier methods.

**Figure 11:**
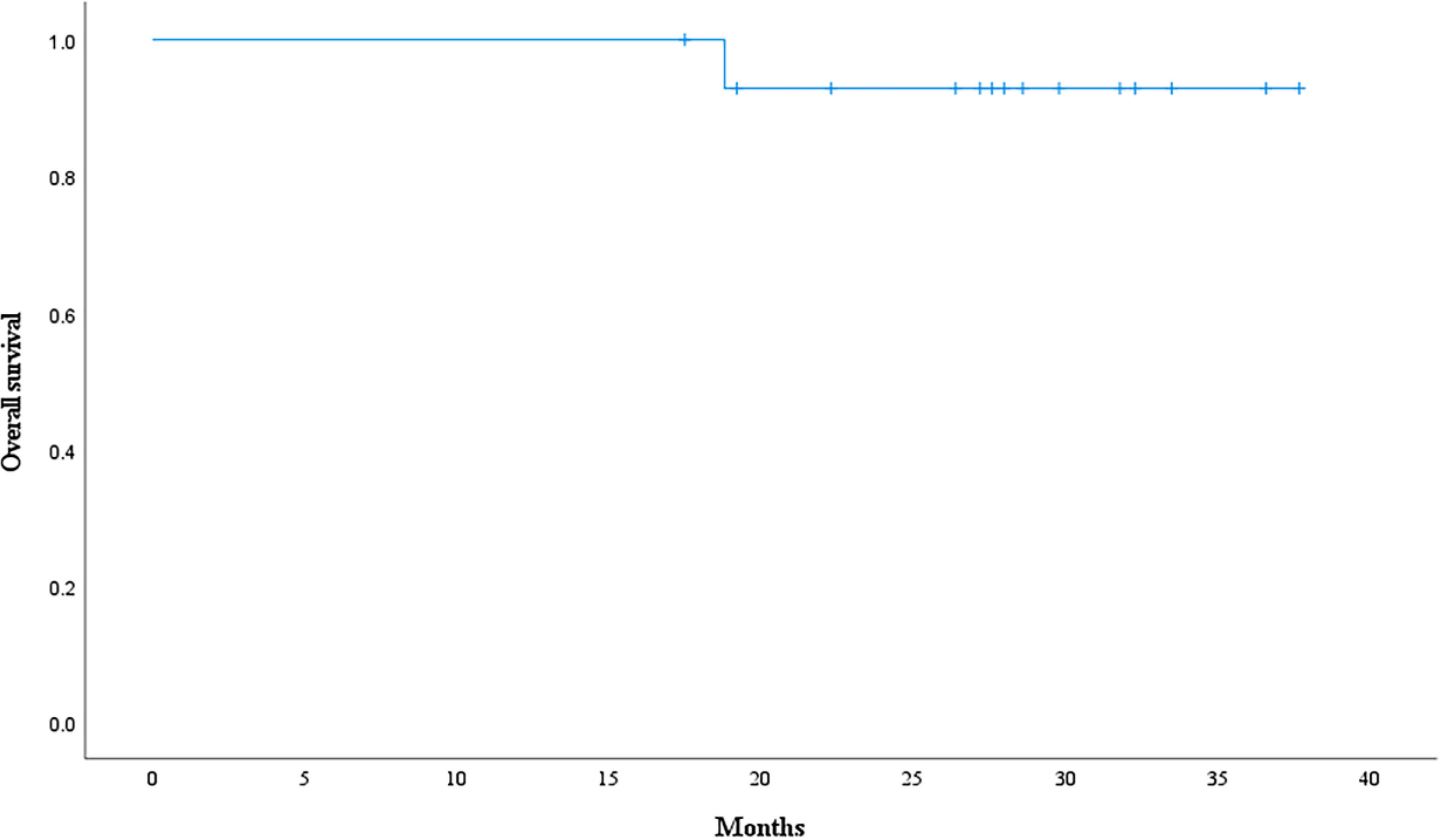
Overall survival curve calculated with Kaplan-Meier methods.

The objective response rate was 91.7% for 12 patients with gross tumor in which 11 patients reached complete response at the first or second tumor evaluation. The median follow-up time is 28.0 months. 3 patients experienced tumor recurrence and none of recurrent lesion was in the radiation field. Event-free survival rate and overall survival rate at 3 years were 79.4% (**Figure 10**) and 92.9% (**Figure 11**). Median event-free survival and overall survival were not reached.

## Discussion

This trial revealed the dosimetric benefits of daily ART for cervical cancer over IGRT. Except for bone marrow, oART technology considerably increases target coverage and dramatically lowers OAR doses. oART techniques necessitate workflows and roles to be reorganized along with precise and quick contouring and treatment planning. Because of these requirements, a radiation oncologist, medical physicist, and/or dosimetrist need to be present while using the treatment machines. Because adaption is time-consuming, timeslots could also need to be changed (20, 21).

In patients with rectal cancer and cervical cancer, most simulated adaptive therapies may have been completed in 30- and 15-min treatment windows, respectively (19). The mean duration in this study was 21.22 min. The simulated ART workflow for cervical cancer was completed in 24.4 min to determine the OARs structure and target contours (19). Henke et al. and Lamb et al. reported ART processes of 23 and 24 min, respectively, for abdominal SBRT by using an MRI-guided system (ViewRay, Oakwood Village, OH) (22). In another study, the median total treatment durations for abdominal SBRT were 46 and 62 min for adapt-to-shape–based and adapt-to-position–based workflows (23).

By preserving target coverage and lowering doses to OARs, online ART has the benefit of providing dosimetric gains. To confirm the disease control and fewer complications of online ART in customized treatment, more research is necessary. Four fundamental technologies are needed for ART: quality assurance, replanning, assessment, and imaging (24). Patients are chosen for adaptation based on alterations in the volume of the tumor or physiological changes to OARs, which are frequently difficult to identify without excellent imaging. To calculate the dosage for the existing AR workflows that incorporate kV CBCT (25) or MRI (26), a pseudo CT has to be created. The pseudo CT is produced by deforming the daily CBCT or MRI from the CT simulation. An assignment of the water-air-bone density may then be necessary for the MRI-based ART workflow. Consequently, the accuracy of the dose calculation depends on the uncertainty of the deformable image registration, which needs to be carefully quantified. The viability of CBCT-only or MRI-only treatment planning is still being investigated, despite the fact that deep learning can be used to create a synthetic CT that is required for dose calculation from CBCT or MRI images (27, 28). Because of its superior CT-to-density accuracy, diagnostic-quality FBCT is currently a better image modality for adaptive radiotherapy.

Yoon et al. used the Varian Ethos system to simulate adaptive therapy for head and neck cancer patients. They presented the closest direct comparison of the metrics reported in the present study (29). The average time required for identifying the influencer structure and target contours and for creating an adaptive replan (11.8 and 6.1 min, respectively) was comparable with those reported here for patients with rectal and cervical cancer, although the process was replicated for a completely different treatment site (19).

Numerous studies have examined how different adaptive processes affect the target coverage and dosage of OARs. Oh et al. simulated numerous ART approaches for cervical cancer patients, while doing so they combined offline adaptive replanning once or once a week with or without bone or soft tissue MRI guidance (30). In this study, we used FBCT, which is a modality of considerably higher quality than CBCT and traditional MVCT (31, 32). ART can be completed more rapidly with diagnostic-grade FBCT than with CBCT-based ART, according to the planned CT used in the present study. ART in which MVCT and CBCT are used can reduce several problems, including the deformation correction and HU value correction of CT images (31–33). The typical changes in the D2cc of the rectum, bladder, bowel, and sigmoid colon, as determined from the dose accumulated by using ART approaches, varied from −0.96 to 0.10 Gy compared with the nominal plan. Furthermore, the average V45Gy discrepancies of these organs varied from −3.9% to 6.4%.

In this study, the PTV Dmax of the ART plan decreased by 1.23 Gy, PTV D95 increased by 1.34 Gy, PTV coverage V50 increased by 4.86%, and CTV coverage increased by 3.02%. On comparing oART replanning with daily IGRT alignment by using CBCT images, Qin et al. discovered that oART reduces doses to multiple OARs, including the bladder (D1% decreased 1.6 Gy) and the rectal wall (D5% decreased 0.9 Gy) and increased the average dose to 99% of the CTV (D99% increased by 1.8 Gy) (34). In our study, the D2cc in the colon, rectum, and small intestine decreased by 1.12, 1.29, and 1.24 Gy, respectively. While V10 and V30 in the pelvis increased by 1.27% and 0.56%, respectively. V30 in the left and right femoral heads decreased by 2.82% and 3.41%. Using an MRI linear accelerator, Dunlop et al. evaluated the dosimetric impact of oART on the prostate (35). Additionally, a 0.5% drop in dosages to OARs, such as the V95% to the rectum, was observed in their study. The benefits of replanning may not always yield dosimetric improvement for all OARs, as evidenced by the 3.5% and 0.8% increases in the bladder V95% and bowel D0.01cc, respectively (36). These benefits also depend on several interrelated factors associated with plan reoptimization. However, the results of the present study are consistent with those of previous studies in showing that oART approaches can augment OAR sparing by several centigrays to several grays per fraction, and target coverage by several percent. After physicians gain more clinical experience with these devices, the exact values of the dosimetric effects of ART approaches will become clearer.

Furthermore, factors unique to each patient may substantially affect the quality of CBCT images, which must be high to achieve accurate contour propagation and deformable image registration. Even when the underlying anatomical alteration presents no significant issue, physicians may have to modify outlines because of artifacts in the image.

Most oART is based on transforming CBCT and MRI images into pseudo-CT images for radiotherapy planning design, which increases the time of oART and introduces certain errors. This study is based on the FBCT-linac accelerator, a fan-beam CT that can be directly used in radiotherapy planning design. The use of the FBCT-linac accelerator prominently shortens the time required for oART and improves the dose accuracy. ART plan was accurately implemented and provided dosimetric results with significant advantages in this study. To ensure the accuracy of dose accumulation in fading tumors, understand the principles of target volume adjustment, determine the trigger conditions for online ART, and determine the potential risks of increasing the radiation dose of each KV-FBCT, among other detailed investigations that are still necessary (37, 38).

This study analyzes the usage time of various stages of FBCT-based oART in detail. The target area, OAR modification time, and radiotherapy planning optimization time occupy a considerable proportion. Therefore, to improve clinical efficiency, we collaborated with a third party to develop an artificial intelligence-based system to delineate target areas and OARs automatically for cervical cancer. To improve the treatment time, the conventional 9-field treatment is replaced with the VMAT plan, thereby reducing the treatment time. However, the optimization time of the VMAT plan also increased accordingly. According to the preliminary research results, employing the VMAT program can reduce a certain amount of ART time, and specific research results will be published in the future.

Online ART presents a lot of difficulties. One is the method of calculating dose accumulation, which takes into consideration changes in dosimetry and anatomy and may be reflected with *in vivo* dose monitoring, to determine the actual total dose delivered. Furthermore, the completion of other tasks is delayed and resource costs rise as a result of the critical processes requiring full human interventions and the presence of dedicated staff, such as medical physicists and radiation oncologists (39, 40). Third, as ART spreads outside of high-volume centers, it will become increasingly important to decide how and when to adapt with more quantitative, automated, or assisted approaches in order to reduce variability and ensure that ART is administered correctly and consistently. Lastly, data demonstrating the effectiveness of online ART is essential to justify the additional resources and help direct its wider implementation. Lastly, data demonstrating the effectiveness of online ART is essential for directing its wider implementation and providing justification for the extra resources and equipment needed for the method.

## Conclusions

To sum up, the oART system improved target coverage and significantly decreased the maximum dose to nearby OARs. Session planning duties must be completed throughout each session, which necessitates additional planning for manpower and workflow. During the process, most time was spent on analyzing and changing target contours. Better contour determination has the most substantial potential to reduce the demand on the resource pool of online ART.

## Data Availability

The original contributions presented in the study are included in the article/supplementary material. Further inquiries can be directed to the corresponding authors.
